# The bacteriocin from the prophylactic candidate *Streptococcus suis* 90-1330 is widely distributed across *S*. *suis* isolates and appears encoded in an integrative and conjugative element

**DOI:** 10.1371/journal.pone.0216002

**Published:** 2019-04-30

**Authors:** Yukun Sun, Iva A. Veseli, Katy Vaillancourt, Michel Frenette, Daniel Grenier, Jean-François Pombert

**Affiliations:** 1 Department of Biology, Illinois Institute of Technology, Chicago, IL, United States of America; 2 Groupe de Recherche en Écologie Buccale, Faculté de Médecine Dentaire, Université Laval, Québec, QC, Canada; 3 Centre de Recherche en Infectiologie Porcine et Avicole, Fonds de Recherche du Québec–Nature et Technologies, Québec, QC, Canada; Institut National de la Recherche Agronomique, FRANCE

## Abstract

The Gram-positive α-hemolytic *Streptococcus suis* is a major pathogen in the swine industry and an emerging zoonotic agent that can cause several systemic issues in both pigs and humans. A total of 35 *S*. *suis* serotypes (SS) have been identified and genotyped into > 700 sequence types (ST) by multilocus sequence typing (MLST). Eurasian ST1 isolates are the most virulent of all *S*. *suis* SS2 strains while North American ST25 and ST28 strains display moderate to low/no virulence phenotypes, respectively. Notably, *S*. *suis* 90–1330 is an avirulent Canadian SS2-ST28 isolate producing a lantibiotic bacteriocin with potential prophylactic applications. To investigate the suitability of this strain for such purposes, we sequenced its complete genome using the Illumina and PacBio platforms. The *S*. *suis* 90–1330 bacteriocin was found encoded in a locus cargoed in what appears to be an integrative and conjugative element (ICE). This bacteriocin locus was also found to be widely distributed across several streptococcal species and in a few *Staphylococcus aureus* strains. Because the locus also confers protection from the bacteriocin, the potential prophylactic benefits of using this strain may prove limited due to the spread of the resistance to its effects. Furthermore, the *S*. *suis* 90–1330 genome was found to code for genes involved in blood survival, suggesting that strain may not be a benign as previously thought.

## Introduction

The quick rise of antibiotics resistance in the microbial world is problematic to multiple fields, including the food industry. As such, novel antibiotic compounds and alternate strategies to treat infections and prevent growth of pathogenic bacterial species are increasingly being sought after. One promising avenue is the prophylactic use of bacteriocin-producing commensal or neutral species as probiotics to outcompete virulent ones [1]. Bacteriocins form a wide range of antimicrobial peptides produced by microorganisms [2]. These molecules, frequently short peptides, can affect a narrow to large spectrum of bacteria excluding the species producing the compounds, which are immune to their effects via varying mechanisms [3]. Because of the potentially disruptive effects on the microflora of the host, the ideal species for prophylactic use would produce compounds whose spectrum do not disrupt the commensal organisms inhabiting the targeted environment. Recently, a nisin-related lantibiotic bacteriocin with a membrane permeabilization activity has been found in *Streptococcus suis* strain 90–1330 [4]. This lanthionine-containing bacteriocin is a killing peptide active against a number of Gram-positive streptococcal and staphylococcal species but shows little to no activity against Gram-negative bacteria [4].

*S*. *suis* is a genetically diverse Gram-positive streptococcal species displaying at least 35 distinct serotypes and categorized in over 700 sequence types by multilocus sequence typing (MLST) [5]. Of these, serotype 2 (SS2) strains are of particular importance to the swine industry, where they can cause a plethora of severe infections in pigs including endocarditis, meningitis, pneumonia and septicemia [6,7]. *S*. *suis* SS2 species are also a growing cause of concern in human health and have been associated with several zoonotic infections [7–16]. Not all serotype 2 species display the same levels of virulence, however; sequence type 1 (ST1) isolates are usually the most virulent while sequence types 25 (ST25) and 28 (ST28) show mild to low/no virulence, respectively [17]. Notably, the bacteriocin-producing *S*. *suis* 90–1330 (aka 1330) is a SS2-ST28 strain that has been established as avirulent in mice and swine [18]. Considering this lack of virulence and the antibacterial activity of its bacteriocin against Gram-positive swine pathogens, including *S*. *suis* SS2-ST1 strains and staphylococcal species *Staphylococcus aureus* and *Staphylococcus hyicus* [4], this bacterium appears to be an excellent candidate for prophylactic use [2] in the swine industry.

Here we sequenced the complete genome of *S*. *suis* 90–1330 using the Illumina and PacBio short and long-read platforms, respectively, to investigate its suitability for prophylactic use and ensure that this strain is indeed devoid of toxin-encoding genes or any other component that could potentially cause problems with its usage as a probiotic. We also explored the origin and distribution of the bacteriocin encoded by *S*. *suis* 90–1330 across streptococcal species. In the process, we used the underlying genomic data to identify which *S*. *suis* strains are the closest to this SS2-ST28 representative.

## Material and methods

### Bacterial culture and DNA purification

*S*. *suis* isolate 90–1330 (aka 1330), originally isolated from pigs, was acquired from Dr. Marcelo Gottschalk, Faculty of Veterinary Medicine, Université de Montréal [18]. The isolate was cultivated at 37°C in THB medium (IBI Scientific, Peosta, IA, USA) with low agitation (100 rpm). Total genomic DNA was purified using the MasterPure Gram Positive DNA Purification Kit (Epicentre Biotechnologies, Madison, WI, USA) with a lysis incubation time of 3 hours and additional centrifugation steps to remove protein precipitates. DNA thus harvested was quantified with a Qubit 2.0 fluorometer (Life Technologies, Waltham, MA, USA) and its purity assessed electrophoretically on 0.8% agarose gels and spectrophotometrically for the presence of RNA and protein contaminants with the A260/A230 and A260/A280 ratios, respectively, using a Nanodrop 2000c (Thermo Scientific, Waltham, MA, USA).

### Genome sequencing

Genome sequencing was performed using the Pacific Biosciences (PacBio) long read and the Illumina short read platforms. PacBio libraries were prepared and sequenced by the University of Michigan DNA Sequencing Core (Ann Arbor, MI, USA). PacBio SMRTbell libraries were prepared per the PacBio 20 Kb+ protocol with g-TUBE shearing (Covaris, Woburn, Massachusetts, USA) and BluePippin (Sage Science, Beverly, MA, USA) size selection. A total of 108,170 polymerase reads (N50: 19.39 Kb) were sequenced using the P6-C4 chemistry for a total of 1.34 Gbp (420x coverage). Illumina libraries were prepared and sequenced by the Plate-forme d'Analyses Génomiques de l'Université Laval (Quebec City, QC, Canada). DNA for Illumina sequencing was fragmented with a Covaris sonicator and libraries were generated using the Illumina TruSeq chemistry. A total of 663,635 Paired-Ends (PE) 300 bp-long reads (1,327,270 reads total) were sequenced on the Illumina MiSeq platform for a total of 398 Mbp (185x coverage).

### Genome assembly

PacBio reads were processed and assembled with the RS_HGAP_Assembly.3 pipeline as implemented in SMRT Analysis 2.3.0 [https://github.com/PacificBiosciences/SMRT-Analysis/wiki/SMRT-Analysis-Software-Installation-v2.3.0]. Base calling on the initial genome assembly was verified by mapping the PacBio reads back onto the assembled genome using the RS_Resequencing.1 pipeline from SMRT Portal. Base calling was further verified and corrected by mapping the Illumina reads onto the refined assembly using Geneious R7.1.8 (Biomatters, Auckland, New Zealand) as follows: reads were mapped using the built-in Map to Reference function, divergent positions were highlighted with the Find Variations/SNPs tool, and bases were corrected accordingly. The genome was circularized by detecting overlaps with BLASTN homology searches [19] and by adjusting the edges with Consed 29.0 [20]. Genome methylation patterns on the final assembly were detected using the RS_Modification_and_Motif_Analysis pipeline in SMRT Portal. The presence of 5-methylcytosine (5mC) was not assessed due to the small kinetic differences involved between methylated and non-methylated bases, as 5mC residues were not converted to 5-carboxylcytosine (5caC) using Tet1-oxidation prior to sequencing.

### Genome annotation and comparative genomic analyses

Transfer and ribosomal RNAs were positioned on the *S*. *suis* 90–1330 genome with tRNAscan-SE 1.3.1 [21] and RNAmmer 1.2 [22], respectively. Preliminary protein predictions and annotations were performed with PROKKA 1.11 [23] with products inferred using an *E*-value threshold of 1e-30 and validated by InterProScan 5.15–54.0 [24] analyses, with conflicting results resolved by manual curation against the UniProt database [25]. Final annotations in the RefSeq genome were performed with the NCBI Prokaryotic Genome Annotation Pipeline (April 2017 release). ComRS competence genes and promoters were further identified by BLAST homology searches using homologous sequences from other *S*. *suis* strains as queries [26,27]. GC-skew analyses were performed with GenSkew [http://genskew.csb.univie.ac.at/]. KEGG pathway categories for each putative protein were attributed with BlastKOALA [http://www.kegg.jp/blastkoala/]. Physical maps were plotted with Circos [28]. Comparative alignments between *S*. *suis* 90–1330 and other genomes were performed using the progressive Mauve algorithm as implemented in Mauve 2.4.0 [29]. Transposons shared between *S*. *suis* 90–1330 and other strains were identified by BLASTP homology searches. Repeated loci were identified using RepeatFinder from the Geneious R9.1.7 package, with ribosomal RNAs filtered out from the results manually.

### Genetic diversity inferences

**Fig 1:**
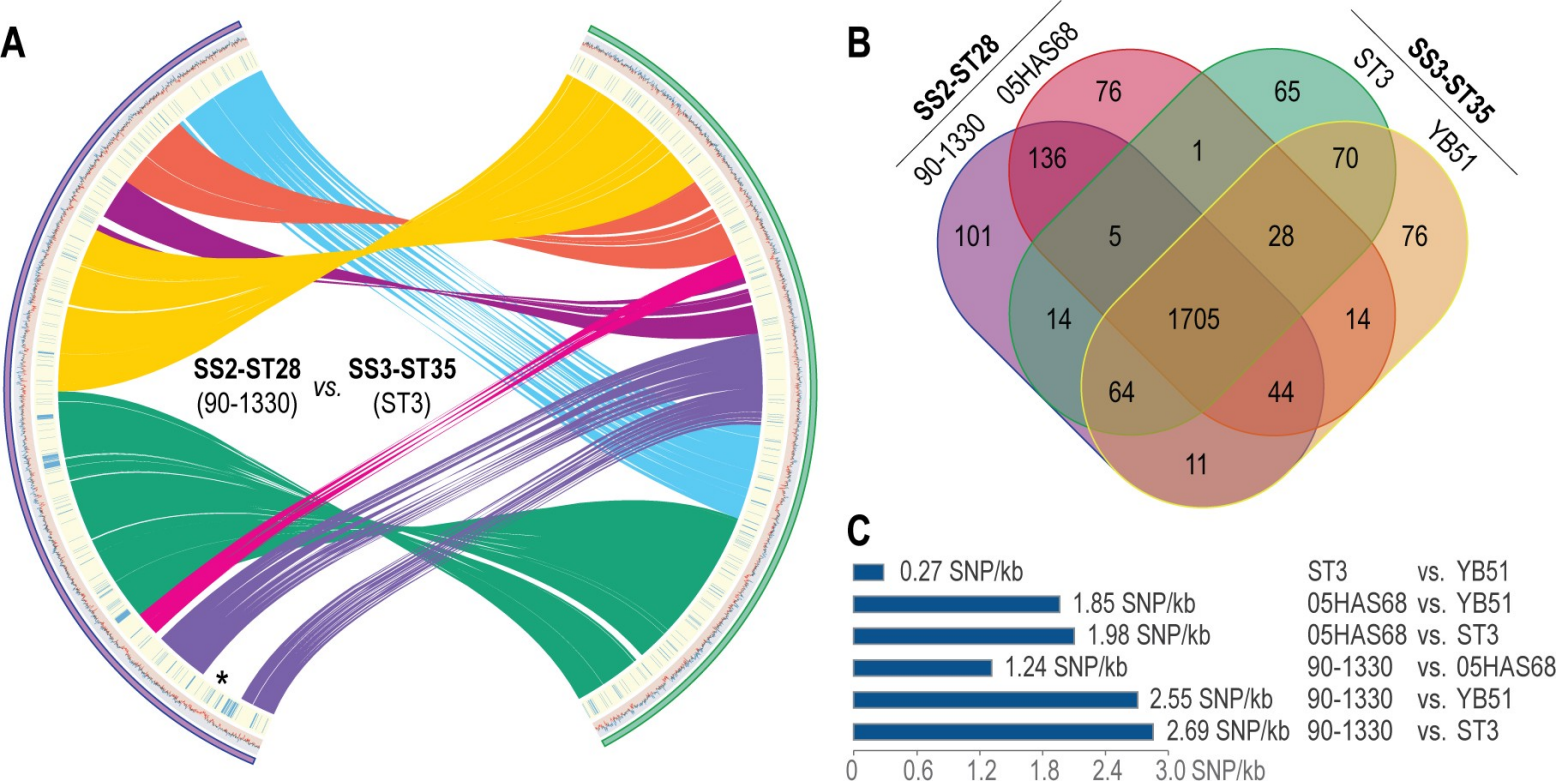
Structural, coding and genetic diversity between *S*. *suis* SS2-ST28 and SS3-ST35 strains. **(A)** Structural genome reorganization between *S*. *suis* 90–1330 (left) and ST3 (right), an SS3-ST35 representative. Syntenic regions that are conserved between 90–1330 and ST3 are highlighted by ribbons. G+C percentages are plotted under each genotype (90–1330, purple; ST3, green), with values higher and lower than 45% and 38% colored in blue and red, respectively. SNPs between the SS2-ST28 strains 90–1330 and 05HAS68 (left) and the SS3-ST35 strains ST3 and YB51 (right) are highlighted by blue bars under the corresponding genotypes. The putative ICE insertion site in 90–1330 is denoted by an asterisk. **(B)** Protein orthologs shared between representative SS2-ST28 and SS3-ST35 strains as identified by BLAST bidirectional best hit approaches (*E*-value cutoff of 1e-10). **(C)** Numbers of pairwise SNPs between representative SS2-ST28 and SS3-ST35 strains.

Complete and draft *S*. *suis* genomes were downloaded from the NCBI GenBank database with the queryNCBI.pl custom Perl script. Pairwise mutation distances using the MinHash dimensionality-reduction technique were calculated with Mash [30] and genetic clusters were plotted with t-Distributed Stochastic Neighbor Embedding (t-SNE [31]) as implemented in the R [32] Rtsne package [33] (https://github.com/jkrijthe/Rtsne) with the run_Mash.pl, MashToDistanceCSV.pl and MashR_plotter.pl custom Perl scripts. Single nucleotide polymorphisms (SNPs) from Fig 1C were inferred using read mapping followed by variant calling. To standardize datasets, synthetic Illumina reads (300 bp paired-ends, insert size 500 bp) were generated for every genome investigated at a sequencing depth of 100X using random subsampling with SSRG.pl 1.5 (https://github.com/PombertLab/SNPs/tree/master/SSRG). Briefly, this Perl script deconstructs a genome (string) into reads (substrings) of specified length in both forward and reverse-complemented orientations using random subsampling, producing synthetic reads akin to reads produced by shotgun sequencing methods. Synthetic reads were mapped against genomes in paired end mode (PE) with Bowtie2 2.3.4.1 [34] and pairwise SNPs were calculated with VarScan2 2.4.3 [35], as implemented in get_SNPs.pl 1.8 (https://github.com/PombertLab/SNPs/tree/master/SSRG) using default parameters. Results from synthetic reads were validated by mapping the *S*. *suis* 90–1330 illumina sequencing data against the same genomes.

### Virulence/antimicrobial resistance predictions, prophage and ice analyses

Streptococcal virulence proteins described by Segura *et al*. [36] were searched for against the *S*. *suis* 90–1330 inferred proteome using BLASTP homology searches with an *E*-value threshold of 1e-10 [19]. Other putative virulence proteins were downloaded from the core dataset of the virulence factor database (VFDB) (http://www.mgc.ac.cn/VFs/main.htm) and searched against *S*. *suis* 90–1330 using BLASTP (*E*-value cutoff: 1e-10). The presence of antimicrobial resistance proteins in *S*. *suis* 90–1330 was assessed using the ResFinder 2.1 online database [37]. Putative prophages were identified using PHASTER [38].

The presence of a putative ICE in *S*. *suis* 90–1330 surrounding the suicin-containing locus was first delimited manually based on the differences highlighted by GC-skew analyses, on the presence of a site-specific integrase, on the presence of ICESa2603-like direct repeats (TTATTTAAGAGTAAC; inferred from [39]) flanking the corresponding locus, on the presence of conjugation proteins within the locus, and on the presence of a putative ICE *ori*T site. Tn*5252* transposon sequences from *Streptococcus pneumoniae* were downloaded from GenBank (accessions numbers L29324.1 and AF295925.1; [40,41]) and searched for against the putative *S*. *suis* 90–1330 ICE using BLASTP searches (*E*-value cutoff: 1e-10). Type IV secretion system proteins were then searched for using CONJscan and TXSScan as implemented in MacSyFinder 1.05 [42,43]. Orthologous proteins between the *S*. *suis* 90–1330 putative ICE and ICESsuCZ130302 from *S*. *suis* CZ130302 (accession number NZ_CP012731.1) were inferred by BLASTP homology searches (*E*-value cutoff: 1e-10). The presence of the integrated and circular forms of the putative ICE in the *S*. *suis* 90–1330 stationary and logarithmic growth phases was assessed by polymerase chain reaction (PCR) with the following primers: GACTCATGCCAAGCCCGAATAG (AN924_RS09900; 1,979,668 to 1,979,689 [forward]), TCTCAGACATAGCCATGCATCC (AN924_RS09905; 1,980,323 to 1,980,339 [reverse complement]), CGCGTAGGCTACCTTAACTTCC (AN924_RS10235; 2,050,558 to 2,050,579 [forward]) and GTTGCATACGCTGTCAAAGCTG (AN924_RS10250; 2,051,504 to 2,051,525 [reverse complement]). PCRs were performed with the following cycles: denaturation (94°C, 1 min), annealing (94°C, 1 min), elongation (72°C, 2 min, 30 cycles), final extension (72°C, 3 min).

## Results

### A normal streptococcal genome

The *S*. *suis* 90–1330 complete genome maps as a single circular molecule of 2,146,151 bp with a GC content of 41.1%. The genome encodes a total of 2,192 genes including 2,120, 56 and 12 genes coding for proteins, transfer RNAs and ribosomal RNAs, respectively. A total of 8 copies of the LSU/SSU ribosomal RNA operon are located on the *S*. *suis* 90–1330 chromosome. The genome has a gene density of 1.13 gene per kb and an average length of 111.5 bp for the intergenic spacers. No gene was found interrupted by group I or group II bacterial introns. The genome features a single bidirectional origin of replication, located at position 1,057,979 of the assembled chromosome (S1 Fig), as calculated by GC-skew analyses and by identifying the position of the *dnaA* gene [44]. A putative mobile genetic element (MGE) featuring a GC content lower by about 10% was also found inserted in the *S*. *suis* 90–1330 chromosome, near one of the two junctions where the GC-skew shifts (S1 Fig). The chromosome encodes several transposable elements that are commonly found in other streptococcal species, including *S*. *suis* ST28 strain 05HAS68 (S2 Table). Only one type (type II) of clustered regularly interspaced short palindromic repeats (CRISPR) was identified, limited to a single locus composed of eight 36 bp-long repeat units separated by unevenly-sized spacers. This locus—from 189,953 to 190,448 on the chromosome—is found in other *Streptococcus* species including strains of *S*. *macedonicus* and *S*. *thermophilus* (from 76% to 89% identity), and displays homology against the genome of *Streptococcus* phage 128 (*E*-value: 2E-18) [45]. The 90–1330 genome also features a few additional dispersed repeated elements, mostly from gene duplicates, with a total of 104 distinct motifs of a least 100 nt distributed across 210 loci, with 16 and 32 repeats longer than 1,000 and 500 nt, respectively (excluding the ribosomal RNA copies; S3 Table). Methylation patterns AGCNNNNNGCT and GTAC were found compatible with the type I and type II restriction–modification systems commonly found in streptococcal species ([46,47]; S1A Fig). In addition to the chromosome, *S*. *suis* strain 90–1330 also contains a 4,984 bp-long circular plasmid (pSS90-1330) encoding a total of 6 genes involved primarily in recombination and replication (S1B Fig).

**Fig 2:**
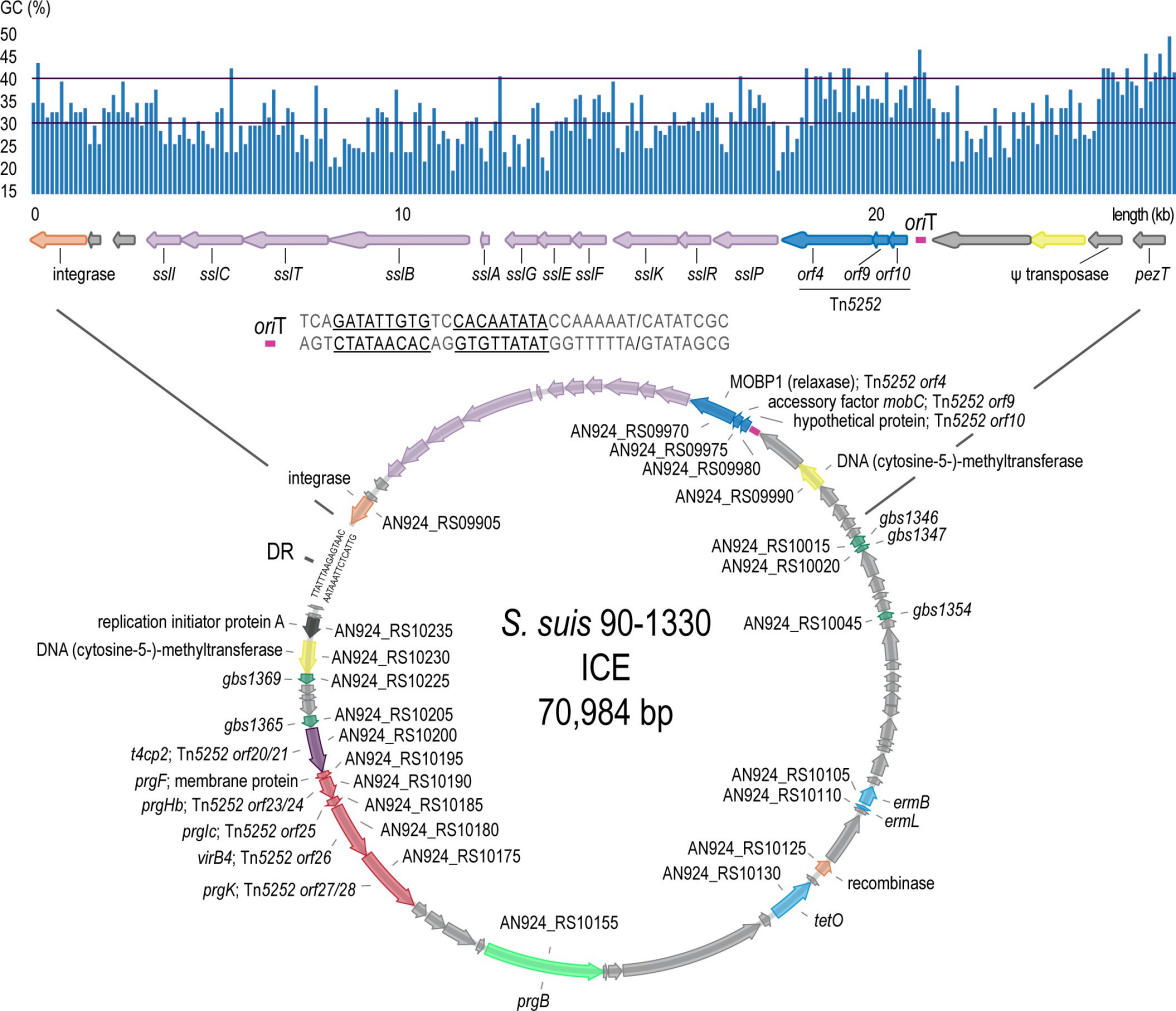
Structure of the putative suicin-containing ICE in *S*. *suis* 90–1330. A zoom-in of the low-GC locus surrounding the bacteriocin and the predicted map of the excised ICE are shown on the top and bottom of the figure, respectively. Genes and their directionality are indicated by arrows and color-coded by their products: orange–integrase/recombinase; light-purple–suicin-related proteins; deep-blue–conjugative DNA processing proteins; deep-purple–Type IV coupling protein; red–Type IV secretion channel proteins; light green–surface adhesin; yellow–DNA methyltransferases; light-blue–antibiotic resistance proteins; deep-gray–replication initiator protein; deep-green–TXSScan FATA_gbs matches (*E*-value cutoff 1e-40); light-gray–miscellaneous. The GC content of the zoom-in is shown in the histogram above the schema (sliding window 100 nt, step 100 nt). *ori*T (origin of transfer); DR (ICESa2603-like direct repeat).

**Fig 3:**
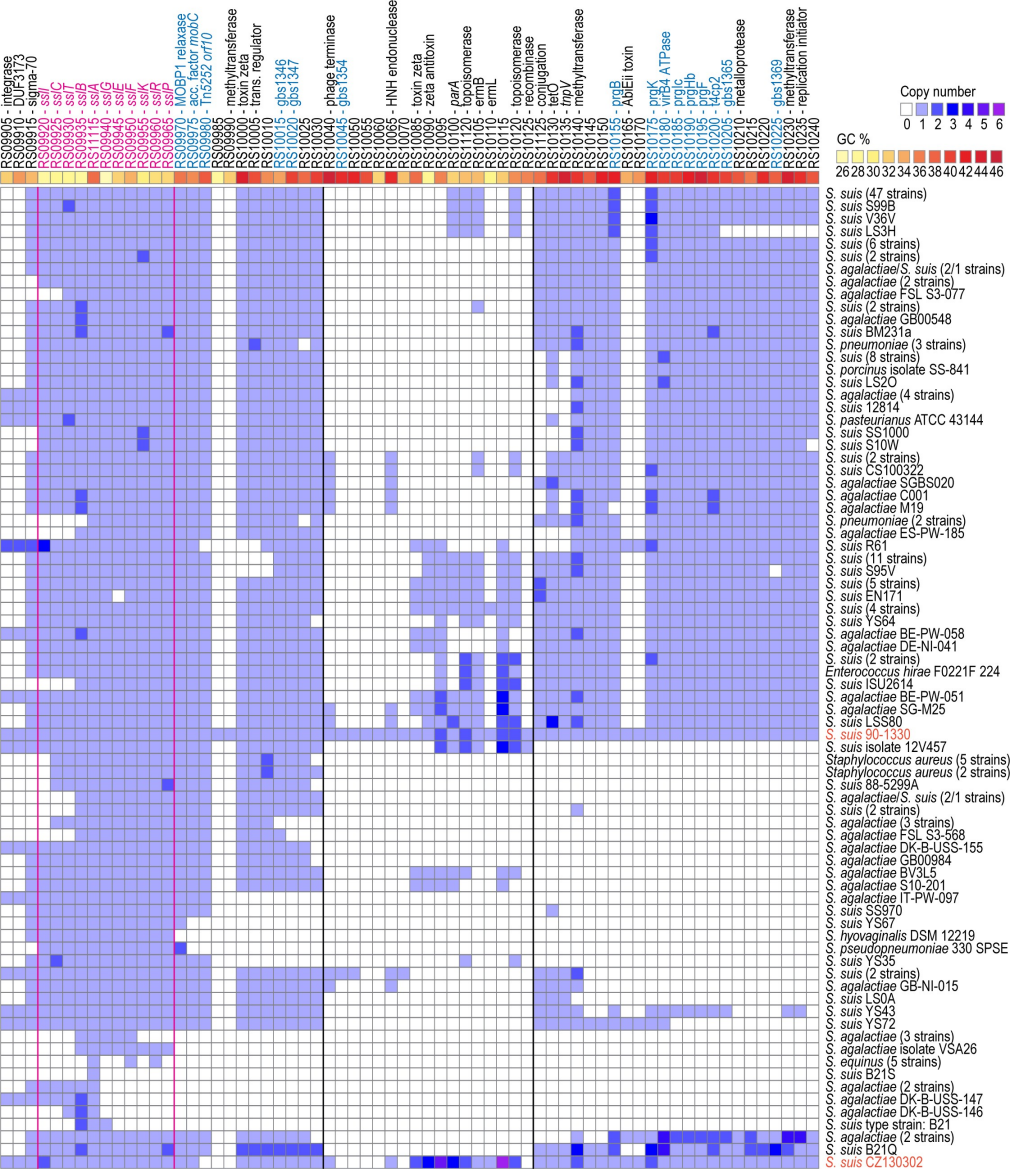
Distribution of the *S*. *suis* 90–1330 suicin-containing locus in other bacterial species. Locus tags/gene names from *S*. *suis* 90–1330 are indicated on top (pseudogenes are not shown); genes from Lebel *et al*. [4] are highlighted in magenta (*sslA;* bacteriocin). ICE-related genes identified by TXSScan/CONJscan searches and by Tn*5252* BLASTP homology searches are highlighted in blue. The internal cargo-like segment of *S*. *suis* 90–1330 is delineated by black bars. Gene copy numbers were inferred by TBLASTN searches with *E*-value and identity cutoffs of 1e-10 and 75%, respectively. Strains with identical contents were collapsed into single rows to increase readability. *S*. *suis* 90–1330 and CZ130302 from S3 Fig are highlighted in orange.

Of the 2,120 protein coding genes found on the *S*. *suis* 90–1330 chromosome, 1,803 were assigned putative functions by the NCBI PGAP pipeline, with 1,161 assigned KEGG orthologs distributed across common streptococcal metabolic pathways (S1 Fig, S4 Table). The *S*. *suis* 90–1330 genome further codes for the type III ComRS competence system typically found in *S*. *suis* genomes, including the methylase DpnA required to prevent degradation of foreign DNA acquired via competence ([26,27,46]; S5 Table), and overall the *S*. *suis* 90–1330 gene content was found very similar to other *S*. *suis* SS2-ST28 genomes, as expected. Interestingly, however, the genotypes of *S*. *suis* SS2-ST28 isolates were found to be nearly identical to those from serotype 3 strains: 1) the SS2-ST28 and SS3-ST35 strains display very high levels of synteny, with only a few chromosomal rearrangements between SS2-ST28 strain 90–1330 and SS3-ST35 strain ST3 (Fig 1A), 2) they share a very large number of genes (Fig 1B), and 3) they show very little divergence at the nucleotide level (Fig 1C). This association between SS2-ST28 and SS3-ST35 strains was found compatible with the previously reported phylogenetic analysis of core *S*. *suis* genes (see figure S4 from Okura *et al*. [48]), in which these strains clustered together. Altogether, these observations suggest that serotype conversion occurred during the evolution of these strains.

### The *S*. *suis* 90–1330 suicin appears encoded in an integrative and conjugative element

The *S*. *suis* 90–1330 bacteriocin AN924_RS09965 (aka suicin [4]) is encoded in a locus displaying an unexpectedly low GC content that contrasts sharply with the remainder of the chromosome (see Fig 2 and note on S1 Fig). In addition to the suicin cluster, this low GC content region contains a gene coding for a conjugative relaxase (AN924_RS09970) and a plasmid mobilization relaxosome protein (AN924_RS09975), both involved in plasmid conjugation [49]. The locus is flanked on either side by a pseudogenized transposase (AN924_RS10000) and a site-specific integrase (AN924_RS09905). The C-terminal segment of the predicted integrase sequence is homologous (*E*-value; 1.65e-38) to tyrosine-based site-specific phage recombinases. However, no other viral gene—including those coding for the viral capsid or tail components and necessary for the lytic cycle—was found in the corresponding locus. Genome-wide searches for the presence of phage sequences in *S*. *suis* 90–1330 revealed four incomplete and one questionable phages with GC contents averaging that of the chromosome (S1 File), none overlapping with the bacteriocin-containing locus.

Besides the abovementioned relaxase (AN924_RS09970) and relaxosome protein (AN924_RS09975), further investigation of the genomic surroundings of the bacteriocin-containing locus revealed the presence of genes coding for a Type IV coupling protein (T4CP; AN924_RS10200), for Type IV secretion system (T4SS) proteins, including a Virb4 ATPase (AN924_RS10180) and a murine hydrolase (AN924_RS10175), and for a surface adhesin (AN924_RS10155; Fig 2; S6 Table) common to integrative and conjugative elements (ICE) [50]. In total, 13 genes from the Firmicutes, Actinobacteria, Tenericutes and Archaea (FATA) and Conjugation (CONJ) sets of hidden Markov models [42] were identified by TXSScan/CONJscan searches (S6 Table). BlastP searches performed in parallel using Tn*5252* transposon sequences from *Streptococcus pneumoniae* [40,41] revealed homology for Tn*5252* open reading frames *orf4* [*E*-value 4e-150], *orf9* [*E*-value 5e-47], *orf10* [*E*-value 6e-33], *orf25* [*E*-value 7e-42], *orf26* [*E*-value 0.0] and for fused open reading frames *orf20/21* [*E*-values 3e-19/0.0], *orf23/24* [*E*-values 4e-45/9e-43] and *orf27/28* [*E*-values 5e-133/0.0] (Fig 2). Conserved domain database (CDD) searches on the site-specific integrase also retrieved homology [*E*-value 1.65e-38] with the C-terminal catalytic domain of integrases from the ICEBs1 family. A putative *ori*T sequence (TCAGATATTGTGTCCACAATATACCAAAAAT/CATATCGC) was found from 1998605 to 1998636 downstream of the gene coding for the accessory relaxosome protein MobC (Tn*5252 orf9*). Two direct repeats (TTATTTAAGAGTAAC) identical in sequence to those from several ICESa2603 family members present in *S*. *suis* and *Streptococcus agalactiae* [51] were found located respectively 45 bp upstream of the 3’end of the integrase, from 1,980,062 to 1,980,076, and downstream of the 3’ end of the gene coding for ribosomal protein L7/L12 (AN924_RS10245), from 2,051,047 to 2,051,061. Notably, the macrolide and tetracycline resistance genes *erm*B and *tet*O were also found encoded in some of these ICESa2603 family members.

To assess the functionality of the putative *S*. *suis* 90–1330 ICE, PCR were performed to investigate the presence of the excised circular form of the ICE (S2 Fig). Results showed the presence of this circular form, confirming the ability of the ICE to adopt the extrachromosomal form that is required for conjugation. This circular form was mainly detected in stationary phase cells while it was barely detected in cells in the logarithmic growth phase (results not shown). A PCR product corresponding to the junction of the genes contiguous to the ICESa2603-like direct repeats was also detected confirming the absence of the chromosome-integrated ICE in a portion of the cells. This type of population heterogeneity has been previously reported for other ICE (*e*.*g*. ICE*Ssu*CZ130302 [52]). Altogether, these results strongly suggest that the bacteriocin cluster is located in an integrative and conjugative element.

Recently, Pan and colleagues characterized an ICE in *S*. *suis* strain CZ130302 (ICE*Ssu*CZ130302) featuring a bacteriocin [52]. Comparisons between the *S*. *suis* 90–1330 suicin locus and that from ICE*Ssu*CZ130302 revealed that the encoded products are nearly identical in sequence (S7 Table), with the bacteriocins differing in sequence by a single amino acid. Further comparisons between the *S*. *suis* 90–1330 putative ICE and ICE*Ssu*CZ130302 revealed that they are also very similar in structure, with most of the differences located in the cargo area of the ICE (S3 Fig).

### The bacteriocin-containing locus is found across several divergent *S*. *suis* isolates

Initially thought to be rare [4], the suicin encoded in *S*. *suis* 90–1330 (AN924_RS11115) was found at 100% amino acid identity in several strains of *S*. *suis*, *S*. *agalactiae*, *Streptococcus pneumoniae* and other streptococcal species, in seven *Staphylococcus aureus* isolates, and in one strain (F0221F 224) of *Enterococcus hirae* (Fig 3). When lowering the similarity threshold to 85%, the bacteriocin was also found in streptococcal species *Streptococcus pseudopneumoniae*, *Streptococcus thoraltensis* and *Streptococcus equinus* (93, 91 and 85% aa identity, respectively). Expanding the search to all genes present in the suicin-containing locus and those contained in the *S*. *suis* 90–1330 putative ICE, a total of 43 genes were found distributed across most of the bacteriocin-containing strains, including ICE*Ssu*CZ130302-encoding *S*. *suis* strain CZ130302 (Fig 3). These genes include the T4SS and conjugative DNA processing proteins identified by TXSScan/CONJscan searches, suggesting that this bacteriocin locus is common in streptococcal ICEs. In most loci, the suicin-containing segments were also found to display lowered GC contents compared to the rest of their surroundings (averaging 31.1% *vs*. 41.1%, respectively). Many strains like *S*. *suis* JS14, *S*. *suis* LS9N or *S*. *agalactiae* C001 feature a different integrase/recombinase than that of *S*. *suis* 90–1330 and the *S*. *suis* 90–1330 suicin locus was found to be closer to that of *Streptococcus pasteurianus* ATCC 43144 (99% identity over 100% of its sequence) than to that of the other *S*. *suis* strains. Interestingly, the inner section of the *S*. *suis* 90–1330 putative ICE appears unique to this genome (Fig 3), with homology searches returning matches covering at best 58% of the region in *Streptococcus parauberis*. The inner segment of the putative ICE element in *S*. *suis* 90–1330 is flanked by a phage terminase and a recombinase, which suggests that the region was added by recombination.

**Fig 4:**
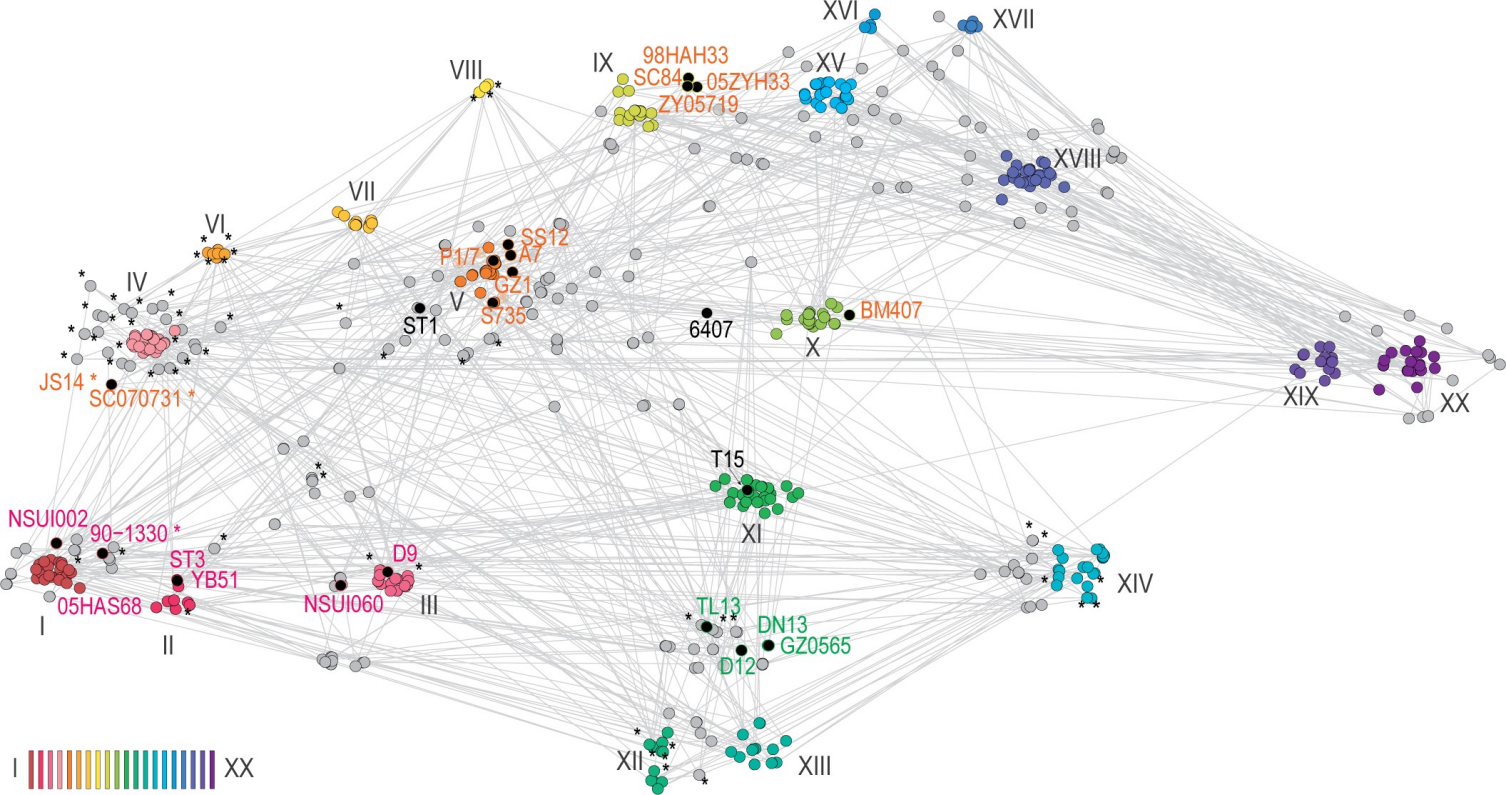
Distribution of the bacteriocin locus across complete and draft *S*. *sui*s genomes (847 total). MASH pairwise genetic distances were clustered by similarity using t-Distributed Stochastic Neighbor Embedding (t-SNE [31]). Bacteriocin-containing clusters are indicated by asterisks (*). Nodes identified by roman numerals (I—XX) were color-coded by proximity. Complete genomes are indicated by black circles and labelled using the corresponding color palette. Note that because of the multidimensional reduction, the relative distances between the nodes are not quite to scale on the two-dimensional plane.

To map the distribution of the bacteriocin-containing locus across *S*. *suis* strains in a phylogenetic framework, we performed genetic distances estimations and plotted their presence on a dimensionality reduction graph. The bacteriocin-containing strains were found scattered across several distinct clusters (I, II, III, IV, V, VIII, XII and XIV; Fig 4), including the highly divergent clusters XII and XIV, suggesting a wide distribution across sequenced *S*. *suis* isolates.

### The *S*. *suis* 90–1330 genome codes for genes involved in blood survival

To investigate the suitability of *S*. *suis* 90–1330 as a prophylactic strain for the swine industry, we searched for the presence of genes coding for virulence factors and for products involved in antimicrobial resistance.

Because there is no clear consensus on what causes pathogenicity in *S*. *suis* strains, we searched the *S*. *suis* 90–1330 genome for all putative virulence factors catalogued and discussed by Segura *et al*. [36] using a permissive cut-off of 1E-10 (see S8 Table). Unsurprisingly, the *S*. *suis* 90–1330 genome codes for capsular polysaccharides (CPS) genes (*e*.*g*. *cpsC*, AN924_RS02950; *cpsE*, AN924_RS02940) and for capsule regulatory genes (*e*.*g*. *ccpA*, AN924_RS01545), as expected from a serotypable strain. In contrast, of the three genes commonly used as multiplex PCR markers to predict *S*. *suis* virulence [5]—*sly* (hemolysin suilysin; a thiol-activated toxin [53]), *epf* (extracellular factor) and *mrp* (muramidase-released protein)—only *mrp* (whose product is involved in epithelial cell adherence [54,55]) was found encoded in the genome. This result was expected based on previous PCR-multiplex virulence inquiries [4] and genomic investigations of various ST28 [56] and ST25 [57] *S*. *suis* strains. Expanding the search to genes coding for products potentially involved in bacteremia and/or enhancing *S*. *suis* survival in blood revealed the presence of *yqfA* (AN924_RS10290) coding for hemolysin III, of *fbpA* (AN924_RS01115) whose product’s N-terminus shows strong homology (9.6E-151) with fibronectin-binding protein A (FbpA) in InterProScan/PFAM searches, and of *iga* (AN924_RS10410), coding for a proteinase potentially involved in host immune evasion [36]. However, the latter was found upon further inspection to be pseudogenized, with read-mapping over the corresponding locus confirming that the gene is indeed broken and not the result of a sequencing error. Overall, only 24 out of the 140 genes searched against the *S*. *suis* 90–1330 genome were not found at the 1E-10 threshold (S8 Table). Additional searches against the virulence factor database (VFDB; http://www.mgc.ac.cn/VFs/main.htm) revealed the presence of more CPS genes and of a gene coding for a TlyC-like (CDD *E*-value 1e-130) hemolysin C family transporter (AN924_RS07935) potentially involved in virulence (S9 Table). These searches also returned a putative match with a cytolysin activator (*cylA*; *E*-value: 8.00E-31) but not for a cytolysin, and upon further inspection the match was found to be spurious, as the corresponding gene (AN924_RS09965) is located on the bacteriocin locus [4] and codes for a lantiobiotic-specific protease (*E*-value 2.81e-114).

As expected from previous work on *S*. *suis* ST28 strains [56,58], *S*. *suis* 90–1330 is not devoid of antimicrobial resistance and the macrolide and tetracycline resistance genes *erm*B/*erm*L and *tet*O were found in the genome with 99.86% and 100% identity against representative sequences from the ResFinder antimicrobial database [37], respectively, over the full length of their coding sequences. Like other streptococcal strains, *S*. *suis* 90–1330 genome codes for several toxin-antitoxin systems (AbiEii/AbiGii; HicA/HicB, RelE/ParE, PezT/PezA, Phd/YefM, Txe/YoeB), and whose overexpression inhibit growth of the bacterium [59]. Interestingly, in addition to the previously reported lantibiotic bacteriocin active against Gram-positive species [4], the *S*. *suis* 90–1330 genome also codes for two Holin (AN924_RS09050, AN924_RS08990) and one Holin-like (AN924_RS10900; pfam16935) toxins with Gram-positive antibacterial properties [60].

## Discussion

In healthy microbiotas, the presence of commensal and/or neutral microorganisms outcompete pathogenic species for the limited pool of nutrients, resources available and adherence sites, providing a helpful barrier against diseases. This helpful association between host and commensal/neutral microorganisms forms the basis behind the prophylactic use of probiotics. Intuitively, prospective probiotics should be devoid of toxicity and side effects while providing clear positive benefits to the health of the individual(s). Recently, strain 90–1330 from the low virulence/nonvirulent *Streptococcus suis* serotype 2 –sequence type 28 was shown to express a bacteriocin with a membrane permeabilization effect that inhibits not only the growth of virulent serotype 2 strains of *S*. *suis*, but also of other Gram positive swine pathogens–including species *hyicus* and *aureus* from the genus *Staphylococcus* [4]. This suggested that its use as a probiotic could serve as a robust preventive method in the swine industry. Here we determined the complete sequence of the *S*. *suis* 90–1330 genome to investigate its suitability as a probiotic. However, our results suggest that the use of this strain for prophylactic purposes may not yield the expected benefits and that despite is apparent lack of virulence [4,18], *S*. *suis* 90–1330 may not be entirely harmless.

### A mobile bacteriocin locus?

One major argument against the use of an unmodified *S*. *suis* 90–1330 strain as a probiotic is the fact that the bacteriocin locus was found encoded within what appears to be an integrative and conjugative element (ICE) shared between different streptococcal species. Mobile genetic elements are common in bacterial genomes and are drivers of genetic diversity between and within lineages [61]. Although derelict/incomplete ICEs are no longer capable of mobility, complete ones can propagate to other loci upon the proper stimuli. The *S*. *suis* 90–1330 bacteriocin locus contained in the putative ICE codes not only for the suicin itself but also provides protection against its effects [4,62]. One can easily envision a scenario wherein a mobile bacteriocin-containing ICE could integrate their genetic material in other microorganisms, including the very pathogens whose growth we are trying to inhibit, under sublethal bacteriocin concentrations. This would render the bacteriocin ineffective against them, and while it is not known exactly how prevalent or efficient this process would be, over time there is a non-negligible probability that an active ICE could integrate itself into the genomes of the very organisms that are we are trying to get rid of. Given that a near identical bacteriocin locus was found in a mobile ICE from *S*. *suis* strain CZ1303002 [52], a potent pathogen shown to cause severe meningitis [63], this scenario likely has occurred in the not so distant past. Furthermore, because Pan and colleagues [52] successfully transconjugated the *S*. *suis* CZ1303002 ICE from suicin+ to suicin- species, it appears that suicin- species can indeed survive long enough to acquire the proper resistance mechanisms under the right conditions.

Here, although the mobility of the *S*. *suis* 90–1330 suicin-containing putative ICE has not been tested *in vitro*, the combined presence of the ICESa2603-like direct repeats, of a putative origin of transfer *ori*T, of an integrase together with the conjugative DNA processing and Type IV secretion system proteins required for the ICE propagation to ectopic sites and recipient cells [61] (Fig 2), together with the detected presence of both integrated and excised forms of this element in *S*. *suis* 90–1330 (S2 Fig), strongly suggests that this element is genuine. Clearly, more experiments are required to confirm that this element is mobile. However, considering that the suicin-containing locus from the *S*. *suis* 90–1330 putative ICE also has been found in the mobile ICE*Ssu*CZ130302 from *S*. *suis* CZ1303002 [52] and in several other bacterial species also harboring ICE-related genes (Fig 3), it would be prudent to consider the *S*. *suis* 90–1330 ICE as capable of mobility unless demonstrated otherwise.

But even if the bacteriocin-containing locus in *S*. *suis* 90–1330 is no longer mobile, streptococcal species are naturally competent [64], and this strain is no exception. All the genes involved in the ComRS competence system and in the methylation of foreign DNA acquired by competence were found in the *S*. *suis* genome, which provides this strain with the opportunity to acquire genetic material from bacteria that it kills with its bacteriocin, and one could envision that by killing pathogenic strains, *S*. *suis* 90–1330 may acquire/reacquire virulence genes. Considering all the above, engineering the *S*. *suis* 90–1330 strain to ensure that the locus is no longer mobile and to knockout the streptococcal competence system prior to its use as a prophylactic appears an essential step to minimize undesirable outcomes.

### Would using *S*. *suis* 90–1330 as a prophylactic provide tangible benefits to the host?

Intuitively, effectiveness and the absence of side effects are desirable traits of any treatment. One of the major draws of using the *S*. *suis* 90–1330 bacteriocin as a substitute for traditional antibiotics resides in its relatively narrow spectrum, which reduces the risk of disturbing the healthy microflora [2]. However, the wide distribution of the *S*. *suis* 90–1330 bacteriocin cluster across several streptococcal strains and species (Figs 3 and 4) raises doubt about the overall effectiveness of using this strain as a prophylactic. Because the bacteriocin locus also confers protection against its effect, many strains are already protected against this bacteriocin, including several pathogens. But even for other streptococcal pathogens lacking this locus, because they are naturally competent, merely knocking out mobility of the *S*. *suis* 90–1330 bacteriocin-containing locus may not be sufficient to prevent its propagation to other organisms. Any genetic material left in the environment by *S*. *suis* 90–1330 could be integrated by pathogens and, therefore, decoupling the bacteriocin from its protectin would help minimize the risks of propagation by preventing the simultaneous acquisition of both genes. Clearly, knocking out the protectin gene from the *S*. *suis* 90–1330 genome would render this strain susceptible to its effect, and is therefore not desirable. Considering these issues, administering the bacteriocin alone via other distribution mechanisms may prove more effective than using *S*. *suis* 90–1330 as a probiotic.

### Is *S*. *suis* 90–1330 truly harmless?

Using *S*. *suis* 90–1330 and the bacteriocin it produces for prophylactic purposes appeared a very interesting prospect given its apparent lack of virulence [18]. However, our results raise doubts as to whether this strain is indeed devoid of toxicity. While we did not identify the suilysin typically associated with *S*. *suis* virulence, we identified several genes whose products are involved in blood survival. Indeed, the presence of genes coding for hemolysin III, for a TlyC-like hemolysin transporter and for platelet-binding protein FbpA all support the ability of this strain to survive if not thrive in the bloodstream. Complementation studies involving TlyC turned a nonhemolytic bacteria into an hemolytic one [65] and FbpA is considered an important factor in infective endocarditis, with the *S*. *suis* FbpA featuring both the N-terminal fibrinogen binding domain and the C-terminal DUF814 domain found in *Enterococcus faecalis* homologs shown to bind to immobilized fibronectin [66]. While previous infection experiments performed on mice and pigs with *S*. *suis* 90–1330 did not lead to infection [18], one of the three intravenous replicates performed on pigs led to a transient lameness suggesting that this strain might not be entirely benign. The presence of several CPS genes in this serotypable strain also suggests that it may be able to avoid phagocytosis to some extent, and the conditions performed to test the virulence of *S*. *suis* 90–1330 cannot, by their nature, reproduce all the possible conditions found in the environment. As discussed recently by Segura *et al*. [36], *S*. *suis* infections usually originate from the respiratory tract and the intravenous method sidesteps potentially critical early infection stages of this pathogen. But even if *S*. *suis* 90–1330 is indeed avirulent, synergies between different strains have been observed in blood infections [47]. Those infections included other supposedly low virulence SS2-ST28 strains, such that under the right conditions, it would be reasonable to expect virulence from the 90–1330 strain.

## Conclusions

The intent for this study was to look for the suitability of using *S*. *suis* 90–1330 as a prophylactic strain in the swine industry. However, based on our results, we cannot recommend its use as is without further engineering given the apparent mobility of its lantibiotic bacteriocin locus cargoed in what is likely an integrative and conjugative element. But even if this element is no longer mobile, given the wide distribution of this bacteriocin and its resistance mechanism across several streptococcal species, the use of this *S*. *suis* isolate as a probiotic may provide only limited protection against virulent strains. Furthermore, based on its genetic paraphernalia, the *S*. *suis* 90–1330 strain may not be as benign as previously thought and further testing and/or engineering would be required to ensure the safety of the animals subjected to potential prophylactic treatments involving this strain. Considering all the above, administering the bacteriocin directly as food preservative/supplement using alternate mechanisms that are not reliant on probiotics may prove a better approach to minimize undesirable outcomes, and further work will be required to determine if using this bacteriocin in such a fashion would indeed be effective.

## Supporting information

S1 Fig Physical map of *Streptococcus suis* 90–1330. **(A)** *S*. *suis* 90–1330 chromosome. Genes located on the forward strand are indicated by orange boxes whereas those located on the minus strand are highlighted in yellow. Methylation patterns and GC-skew distribution are indicated in the outer and inner rings, respectively. GC-skew patterns (positive and negative values are shown as red and blue, respectively). The putative origin of replication inferred by the GC-skew analyses and the location of *dnaA* is indicated by an arrow. MGE; bacteriocin-containing mobile genetic element. The sixteen inner rings surrounded by the GC skew plot highlight genes of known function color-coded per KEGG pathways. From outside to inside: amino acid metabolism (orange; 126 genes), carbohydrate metabolism (blue; 246 genes), cellular processes (light green; 14 genes), metabolism of cofactors and vitamins (pink; 57 genes), energy metabolism (violet; 68 genes), environmental information processing (yellow; 151 genes), genetic information processing (pale red; 181 genes), glycan biosynthesis and metabolism (blue; 24 genes), human diseases (green; 50 genes), lipid metabolism (dark cyan, 42 genes), nucleotide metabolism (red; 99 genes), organismal systems (green; 15 genes), metabolism of other amino acids (orange; 26 genes), biosynthesis of other secondary metabolites (pink; 23 genes), metabolism of terpenoids and polyketides (lime green; 24 genes) and xenobiotics biodegradation and metabolism (brown; 22 genes). **(B)** *S*. *suis* 90–1330 plasmid.
(PDF)

S2 Fig Detection of the circular form of the *S*. *suis* 90–1330 ICE. **(A)** Diagram of the chromosomic region contiguous to the ICE integration site. The ICESa2603-like direct repeats are represented by yellow boxes. The orientation of the primers used to detect the integrated and circular forms are indicated by thin arrows (see Material and Methods for the primer sequences): 1) AN924_RS09900 –hypothetical protein; 2) AN924_RS09905 –integrase; 3) AN924_RS10235 –replication initiator protein; 4) AN924_RS10235 –ribosomal protein L7/L12. **(B)** Electrophoretic analysis of the PCR products (0.8% agarose gel); primer pairs are shown above the lanes.
(PDF)

S3 Fig Structural comparison between the *S*. *suis* 90–1330 putative ICE and ICESsuCZ130302 from *S*. *suis* CZ130302. Conserved blocks of genes arrayed in the same order between the two loci are highlighted by alternating colors. Locus tags are derived from accession numbers NZ_CP012731.1 (*S*. *suis* 90–1330) and NZ_CP024974.1 (*S*. *suis* CZ130302).
(PDF)

S1 File Putative prophages in the *S*. *suis* 90–1330 genomes. This file is the raw output from PHASTER.
(TXT)

S1 Table *S*. *suis* strains used in this study. Predicted serotypes are based on BLASTN searches using the *cps* genes from Liu *et al*. [67] against the *S*. *suis* accession numbers.
(XLSX)

S2 Table Predicted transposable elements in *S*. *suis* 90–1330. Transposable elements listed in S1 Table were derived from the NCBI PGAP annotations.
(XLSX)

S3 Table *S*. *suis* 90–1330 repeat regions. Repeated loci were identified with RepeatFinder as implemented in Geneious R9.1.7.
(XLSX)

S4 Table Distribution of the *S*. *suis* 90–1330 KEGG metabolic pathways. KEGG metabolic pathways and enzyme commission (EC) numbers were assigned with BlastKOALA.
(XLSX)

S5 Table ComRS competence components in *S*. *suis* 90–1330. ComRS proteins and promoters were identified by BLAST searches of known orthologous streptococcal sequences against the *S*. *suis* 90–1330 genome.
(XLSX)

S6 Table ICE proteins identified in the *S*. *suis* 90–1330 putative ICE by CONJscan/TXSScan searches. CONJscan/TXSScan searches were performed with MacSyFinder 1.05. Duplicate hits with lower *E*-values are grayed out.
(XLSX)

S7 Table Orthologs between the *S*. *suis* 90–1330 putative ICE and ICESsuCZ130302. Orthologs were inferred by BLASTP searches (*E*-value cutoff: 1e-10). Genes from Lebel *et al*. [4] are highlighted in magenta (*sslA;* bacteriocin). ICE-related genes identified by TXSScan/CONJscan searches and by Tn*5252* BLASTP homology searches are highlighted in blue.
(XLSX)

S8 Table Putative virulence factor homologs in *S*. *suis* 90–1330. The virulence factors discussed in Segura *et al*. [36] were searched against the *S*. *suis* 90–1330 genome using BLAST searches. Genes potentially important in virulence are highlighted in orange; pseudogenes are grayed out.
(XLSX)

S9 Table Putative additional virulence factors in *S*. *suis* 90–1330 derived from homology searches against the core dataset of the Virulence Factor Database (VFDB). *S*. *suis* proteins displaying homology with putative VFDB virulence factors (E-value cutoff: 1e-10) are listed here.
(XLSX)

## References

[1] Yang S-C, Lin C-H, Sung CT, Fang J-Y. Antibacterial activities of bacteriocins: application in foods and pharmaceuticals. Front Microbiol. Frontiers Media SA; 2014;5: 241 10.3389/fmicb.2014.00241 24904554 PMC4033612

[2] Dobson A, Cotter PD, Ross RP, Hill C. Bacteriocin production: a probiotic trait? Appl Environ Microbiol. American Society for Microbiology; 2012;78: 1–6. 10.1128/AEM.05576-11 22038602 PMC3255625

[3] Cotter PD, Hill C, Ross RP. Food Microbiology: Bacteriocins: developing innate immunity for food. Nat Rev Microbiol. 2005;3: 777–788. 10.1038/nrmicro1273 16205711

[4] LeBel G, Vaillancourt K, Frenette M, Gottschalk M, Grenier D. Suicin 90–1330 from a nonvirulent strain of *Streptococcus suis*: a nisin-related lantibiotic active on gram-positive swine pathogens. Appl Environ Microbiol. 2014;80: 5484–5492. 10.1128/AEM.01055-14 24973067 PMC4136082

[5] King SJ, Leigh JA, Heath PJ, Luque I, Tarradas C, Dowson CG, et al Development of a multilocus sequence typing scheme for the pig pathogen *Streptococcus suis*: Identification of virulent clones and potential capsular serotype exchange. J Clin Microbiol. 2002;40: 3671–3680. 10.1128/JCM.40.10.3671-3680.2002 12354864 PMC130843

[6] Zimmerman JJ. Diseases of swine. Zimmerman JJ, Karrike LA, Alejandro R, Schwartz KJ, Stevenson GW, editors. Wiley-Blackwell; 2012.

[7] Domínguez-Punaro MC, Segura M, Plante M-M, Lacouture S, Rivest S, Gottschalk M. *Streptococcus suis* serotype 2, an important swine and human pathogen, induces strong systemic and cerebral inflammatory responses in a mouse model of infection. J Immunol. 2007;179: 1842–54. 17641051 10.4049/jimmunol.179.3.1842

[8] Dupas D, Vignon M, Géraut C. *Streptococcus suis* meningitis. A severe noncompensated occupational disease. J Occup Med. 1992;34: 1102–5. 1432301

[9] Huong VTL, Long HB, Kinh N V., Ngan TTD, Dung VTV, Nadjm B, et al Long-term outcomes of patients with *Streptococcus suis* infection in Viet Nam: A case-control study. J Infect. 2017; 10.1016/j.jinf.2017.09.019 PMC5790056 28970042

[10] Loetthong P, Anukul R, Tanburawong N, Samerchea S, Areeratana P, Lekhalula P, et al Impact of a Food Safety Campaign on *Streptococcus suis* Infection in Humans in Thailand. Am J Trop Med Hyg. 2017;96: 1370–1377. 10.4269/ajtmh.16-0456 28719258 PMC5462574

[11] Callejo R, Zheng H, Du P, Prieto M, Xu J, Zielinski G, et al *Streptococcus suis* serotype 2 strains isolated in Argentina (South America) are different from those recovered in North America and present a higher risk for humans. JMM Case Reports. 2016;3: e005066 10.1099/jmmcr.0.005066 28348788 PMC5343146

[12] Mancini F, Adamo F, Creti R, Monaco M, Alfarone G, Pantosti A, et al A fatal case of streptococcal toxic shock syndrome caused by *Streptococcus suis* carrying *tet* (40) and *tet* (O/W/32/O), Italy. J Infect Chemother. 2016;22: 774–776. 10.1016/j.jiac.2016.05.011 27553071

[13] Smith TC, Capuano AW, Boese B, Myers KP, Gray GC. Exposure to *Streptococcus suis* among US swine workers. Emerg Infect Dis. 2008;14: 1925–7. 10.3201/eid1412.080162 19046523 PMC2634616

[14] van de Beek D, Spanjaard L, de Gans J. *Streptococcus suis* meningitis in the Netherlands. J Infect. 2008;57: 158–161. 10.1016/j.jinf.2008.04.009 18538852

[15] Tramontana AR, Graham M, Sinickas V, Bak N. An Australian case of *Streptococcus suis* toxic shock syndrome associated with occupational exposure to animal carcasses. Med J Aust. 2008;188: 538–9. 18459929 10.5694/j.1326-5377.2008.tb01771.x

[16] Watkins EJ, Brooksby P, Schweiger MS, Enright SM. Septicaemia in a pig-farm worker. Lancet. 2001;357: 38 11197360 10.1016/s0140-6736(00)03570-4

[17] Fittipaldi N, Xu J, Lacouture S, Tharavichitkul P, Osaki M, Sekizaki T, et al Lineage and virulence of *Streptococcus suis* serotype 2 isolates from North America. Emerg Infect Dis. 2011;17: 2239–2244. 10.3201/eid1712.110609 22172538 PMC3311171

[18] Quessy S, Dubreuil JD, Caya M, Higgins R. Discrimination of virulent and avirulent *Streptococcus suis* capsular type 2 isolates from different geographical origins. Infect Immun. American Society for Microbiology (ASM); 1995;63: 1975–9. 7729910 10.1128/iai.63.5.1975-1979.1995 PMC173252

[19] Camacho C, Coulouris G, Avagyan V, Ma N, Papadopoulos J, Bealer K, et al BLAST+: architecture and applications. BMC Bioinformatics. 2009;10: 421 Artn 421\n 10.1186/1471-2105-10-421 20003500 PMC2803857

[20] Gordon D, Abajian C, Green P. Consed: a graphical tool for sequence finishing. Genome Res. 1998;8: 195–202. 9521923 10.1101/gr.8.3.195

[21] Lowe TM, Eddy SR. tRNAscan-SE: a program for improved detection of transfer RNA genes in genomic sequence. Nucleic Acids Res. 1997;25: 955–964. 9023104 10.1093/nar/25.5.955 PMC146525

[22] Lagesen K, Hallin P, Rødland EA, Staerfeldt H-H, Rognes T, Ussery DW. RNAmmer: consistent and rapid annotation of ribosomal RNA genes. Nucleic Acids Res. 2007;35: 3100–3108. 10.1093/nar/gkm160 17452365 PMC1888812

[23] Seemann T. Prokka: rapid prokaryotic genome annotation. Bioinformatics. 2014;30: 2068–9. 10.1093/bioinformatics/btu153 24642063

[24] Jones P, Binns D, Chang H-Y, Fraser M, Li W, McAnulla C, et al InterProScan 5: genome-scale protein function classification. Bioinformatics. 2014;30: 1236–1240. 10.1093/bioinformatics/btu031 24451626 PMC3998142

[25] The Uniprot Consortium. Activities at the Universal Protein Resource (UniProt). Nucleic Acids Res. 2014;42: D191–D198. 10.1093/nar/gkt1140 24253303 PMC3965022

[26] Talagas A, Fontaine L, Ledesma-Garca L, Mignolet J, Li de la Sierra-Gallay I, Lazar N, et al Structural Insights into Streptococcal Competence Regulation by the Cell-to-Cell Communication System ComRS. PLoS Pathog. 2016;12: e1005980 10.1371/journal.ppat.1005980 27907189 PMC5131891

[27] Zaccaria E, Van Baarlen P, De Greeff A, Morrison DA, Smith H, Wells JM. Control of competence for DNA transformation in *Streptococcus suis* by genetically transferable pherotypes. PLoS One. 2014;9: e99394 10.1371/journal.pone.0099394 24968201 PMC4072589

[28] Krzywinski M, Schein J, Birol I, Connors J, Gascoyne R, Horsman D, et al Circos: an information esthetic for comparative genomics. Genome Res. 2009;19: 1639–1645. 10.1101/gr.092759.109 19541911 PMC2752132

[29] Darling ACE, Mau B, Blattner FR, Perna NT. Mauve: Multiple Alignment of Conserved Genomic Sequence With Rearrangements. Genome Res. 2004;14: 1394–1403. 10.1101/gr.2289704 15231754 PMC442156

[30] Ondov BD, Treangen TJ, Mallonee AB, Bergman NH, Koren S, Phillippy AM. Fast genome and metagenome distance estimation using MinHash. Genome Biol. Genome Biology; 2016;17: 132 10.1186/s13059-016-0997-x 27323842 PMC4915045

[31] Van Der Maaten LJP, Hinton GE. Visualizing high-dimensional data using t-SNE. J Mach Learn Res. 2008;9: 2579–2605. 10.1007/s10479-011-0841-3

[32] R Core Team. R: A Language and Environment for Statistical Computing. R Found Stat Comput. 2016;version 3: 3503 10.1007/978-3-540-74686-7

[33] van der Maaten L. Barnes-Hut-SNE. arxiv.org. 2013; 1301.3342v2.

[34] Langmead B, Salzberg SL. Fast gapped-read alignment with Bowtie 2. Nat Methods. 2012;9: 357–359. 10.1038/nmeth.1923 22388286 PMC3322381

[35] Koboldt DC, Zhang Q, Larson DE, Shen D, McLellan MD, Lin L, et al VarScan 2: somatic mutation and copy number alteration discovery in cancer by exome sequencing. Genome Res. 2012;22: 568–576. 10.1101/gr.129684.111 22300766 PMC3290792

[36] Segura M, Fittipaldi N, Calzas C, Gottschalk M. Critical *Streptococcus suis* Virulence Factors: Are They All Really Critical? Trends Microbiol. Elsevier Ltd; 2017;25: 585–599. 10.1016/j.tim.2017.02.005 28274524

[37] Zankari E, Hasman H, Cosentino S, Vestergaard M, Rasmussen S, Lund O, et al Identification of acquired antimicrobial resistance genes. J Antimicrob Chemother. Oxford University Press; 2012;67: 2640–2644. 10.1093/jac/dks261 22782487 PMC3468078

[38] Arndt D, Grant JR, Marcu A, Sajed T, Pon A, Liang Y, et al PHASTER: a better, faster version of the PHAST phage search tool. Nucleic Acids Res. 2016;44: W16–21. 10.1093/nar/gkw387 27141966 PMC4987931

[39] Huang J, Ma J, Shang K, Hu X, Liang Y, Li D, et al Evolution and Diversity of the Antimicrobial Resistance Associated Mobilome in *Streptococcus suis*: A Probable Mobile Genetic Elements Reservoir for Other Streptococci. Front Cell Infect Microbiol. 2016;6: 1–14. 10.3389/fcimb.2016.00001 27774436 PMC5053989

[40] Kiliç AO, Vijayakumar MN, Al-Khaldi SF. Identification and nucleotide sequence analysis of a transfer-related region in the streptococcal conjugative transposon Tn5252. J Bacteriol. 1994;176: 5145–50. Available: http://www.ncbi.nlm.nih.gov/pubmed/8051031 8051031 10.1128/jb.176.16.5145-5150.1994 PMC196358

[41] Alarcon-Chaidez F, Sampath J, Srinivas P, Vijayakumar M. TN5252: a model for complex streptococcal conjugative transposons. Adv Exp Med Biol. 1997;418: 1029–32. Available: http://www.ncbi.nlm.nih.gov/pubmed/9331826 9331826 10.1007/978-1-4899-1825-3_242

[42] Guglielmini J, Néron B, Abby SS, Garcillán-Barcia MP, la Cruz F de, Rocha EPC. Key components of the eight classes of type IV secretion systems involved in bacterial conjugation or protein secretion. Nucleic Acids Res. 2014;42: 5715–5727. 10.1093/nar/gku194 24623814 PMC4027160

[43] Abby SS, Cury J, Guglielmini J, Néron B, Touchon M, Rocha EPC. Identification of protein secretion systems in bacterial genomes. Sci Rep. Nature Publishing Group; 2016;6: 23080 10.1038/srep23080 26979785 PMC4793230

[44] Rajewska M, Wegrzyn K, Konieczny I. AT-rich region and repeated sequences—the essential elements of replication origins of bacterial replicons. FEMS Microbiol Rev. 2012;36: 408–434. 10.1111/j.1574-6976.2011.00300.x 22092310

[45] Achigar R, Magadán AH, Tremblay DM, Julia Pianzzola M, Moineau S. Phage-host interactions in *Streptococcus thermophilus*: Genome analysis of phages isolated in Uruguay and ectopic spacer acquisition in CRISPR array. Sci Rep. Nature Publishing Group; 2017;7: 43438 10.1038/srep43438 28262818 PMC5338259

[46] De Ste Croix M, Vacca I, Kwun MJ, Ralph JD, Bentley SD, Haigh R, et al Phase-variable methylation and epigenetic regulation by type I restriction-modification systems. FEMS Microbiol Rev. 2017;41: S3–S15. 10.1093/femsre/fux025 28830092

[47] Willemse N, Schultsz C. Distribution of Type I Restriction–Modification Systems in *Streptococcus suis*: An Outlook. Pathogens. 2016;5: 62 10.3390/pathogens5040062 PMC5198162 27869755

[48] Okura M, Nozawa T, Watanabe T, Murase K, Nakagawa I, Takamatsu D, et al A locus encoding variable defense systems against invading DNA identified in *Streptococcus suis*. Genome Biol Evol. 2017;9: 1000–1012. 10.1093/gbe/evx062 PMC5398294 28379509

[49] Goessweiner-mohr N, Arends K, Keller W, Grohmann E. Conjugation in Gram-Positive Bacteria. Microbiol Specturm. 2014;2: PLAS-0004-2013 10.1128/microbiolspec.PLAS-0004 26104193

[50] Alvarez-Martinez CE, Christie PJ. Biological Diversity of Prokaryotic Type IV Secretion Systems. Microbiol Mol Biol Rev. 2009;73: 775–808. 10.1128/MMBR.00023-09 19946141 PMC2786583

[51] Huang J, Liang Y, Guo D, Shang K, Ge L, Kashif J, et al Comparative genomic analysis of the ICES*a*2603 family ICEs and spread of *erm*(B)- and *tet*(O)-carrying transferable 89K-subtype ICEs in swine and bovine isolates in China. Front Microbiol. 2016;7: 1–13. 10.3389/fmicb.2016.00001 26870017 PMC4735348

[52] Pan Z, Liu J, Zhang Y, Chen S, Ma J, Dong W, et al A novel integrative conjugative element mediates transfer of multi-drug resistance between *Streptococcus suis* strains of different serotypes. Vet Microbiol. 2019;229: 110–116. 10.1016/j.vetmic.2018.11.028 30642585

[53] Jacobs AA, Loeffen PL, van den Berg AJ, Storm PK. Identification, purification, and characterization of a thiol-activated hemolysin (suilysin) of *Streptococcus suis*. Infect Immun. 1994;62: 1742–8. 8168935 10.1093/benz/9780199773787.article.b00034458 PMC186398

[54] Smith HE, Vecht U, Gielkens AL, Smits MA. Cloning and nucleotide sequence of the gene encoding the 136-kilodalton surface protein (muramidase-released protein) of *Streptococcus suis* type 2. Infect Immun. American Society for Microbiology (ASM); 1992;60: 2361–7. 1587602 10.1128/iai.60.6.2361-2367.1992 PMC257166

[55] Wang J, Kong D, Zhang S, Jiang H, Zheng Y, Zang Y, et al Interaction of fibrinogen and muramidase-released protein promotes the development of *Streptococcus suis* meningitis. Front Microbiol. 2015;6: 1001 10.3389/fmicb.2015.01001 26441928 PMC4585153

[56] Athey TBT, Auger J-P, Teatero S, Dumesnil A, Takamatsu D, Wasserscheid J, et al Complex population structure and virulence differences among serotype 2 *Streptococcus suis* strains belonging to sequence type 28. PLoS One. 2015;10: e0137760 10.1371/journal.pone.0137760 26375680 PMC4574206

[57] Athey TBT, Teatero S, Takamatsu D, Wasserscheid J, Dewar K, Gottschalk M, et al Population structure and antimicrobial resistance profiles of *Streptococcus suis* serotype 2 sequence type 25 strains. PLoS One. 2016;11: e0150908 10.1371/journal.pone.0150908 26954687 PMC4783015

[58] Palmieri C, Magi G, Mingoia M, Bagnarelli P, Ripa S, Varaldo PE, et al Characterization of a *Streptococcus suis tet*(O/W/32/O)-carrying element transferable to major streptococcal pathogens. Antimicrob Agents Chemother. 2012;56: 4697–702. 10.1128/AAC.00629-12 22710115 PMC3421841

[59] Hall AM, Gollan B, Helaine S. Toxin–antitoxin systems: reversible toxicity. Curr Opin Microbiol. 2017;36: 102–110. 10.1016/j.mib.2017.02.003 28279904

[60] Rajesh T, Anthony T, Saranya S, Pushpam PL, Gunasekaran P. Functional characterization of a new holin-like antibacterial protein coding gene *tmp1* from goat skin surface metagenome. Appl Microbiol Biotechnol. 2011;89: 1061–1073. 10.1007/s00253-010-2907-6 20927512

[61] Johnson CM, Grossman AD. Integrative and Conjugative Elements (ICEs): What They Do and How They Work. Annu Rev Genet. 2015;49: 577–601. Integrative 10.1146/annurev-genet-112414-055018 26473380 PMC5180612

[62] Bierbaum G, Sahl H-G. Lantibiotics: Mode of Action, Biosynthesis and Bioengineering. Curr Pharm Biotechnol. 2009;10: 2–18. 10.2174/138920109787048616 19149587

[63] Pan Z, Ma J, Dong W, Song W, Wang K, Lu C, et al Novel variant serotype of *Streptococcus suis* isolated from piglets with meningitis. Appl Environ Microbiol. American Society for Microbiology; 2015;81: 976–85. 10.1128/AEM.02962-14 25416757 PMC4292476

[64] Tohya M, Watanabe T, Maruyama F, Arai S, Ota A, Athey TBT, et al Comparative genome analyses of *Streptococcus suis* isolates from endocarditis demonstrate persistence of dual phenotypic clones. PLoS One. 2016;11: 1–16. 10.1371/journal.pone.0159558 PMC4951133 27433935

[65] Radulovic S, Troyer JM, Beier MS, Lau AOT, Azad AF. Identification and Molecular Analysis of the Gene Encoding *Rickettsia typhi* Hemolysin. Infect Immun. American Society for Microbiology Journals; 1999;67: 6104–6108. Available: https://iai.asm.org/content/67/11/6104.long 10.1128/iai.67.11.6104-6108.1999 PMC96999 10531273

[66] Singh K V., Rosa SL La, Somarajan SR, Roh JH, Murray BE. The fibronectin-binding protein EfbA contributes to pathogenesis and protects against infective endocarditis caused by *Enterococcus faecalis*. Infect Immun. 2015;83: 4487–4494. 10.1128/IAI.00884-15 26351286 PMC4645374

[67] Liu Z, Zheng H, Gottschalk M, Bai X, Lan R, Ji S, et al Development of Multiplex PCR Assays for the Identification of the 33 Serotypes of *Streptococcus suis*. PLoS One. 2013;8: 1–11. 10.1371/journal.pone.0072070 PMC3739753 23951285

