# Supplementary figures and images for: The bacteriocin from the prophylactic candidate Streptococcus suis 90-1330 is widely distributed across S. suis isolates and appears encoded in an integrative and conjugative element
Source: PLoS One. 2019 Apr 30;14(4):e0216002. doi: 10.1371/journal.pone.0216002 (PMC6490898; doi:10.1371/journal.pone.0216002)

A

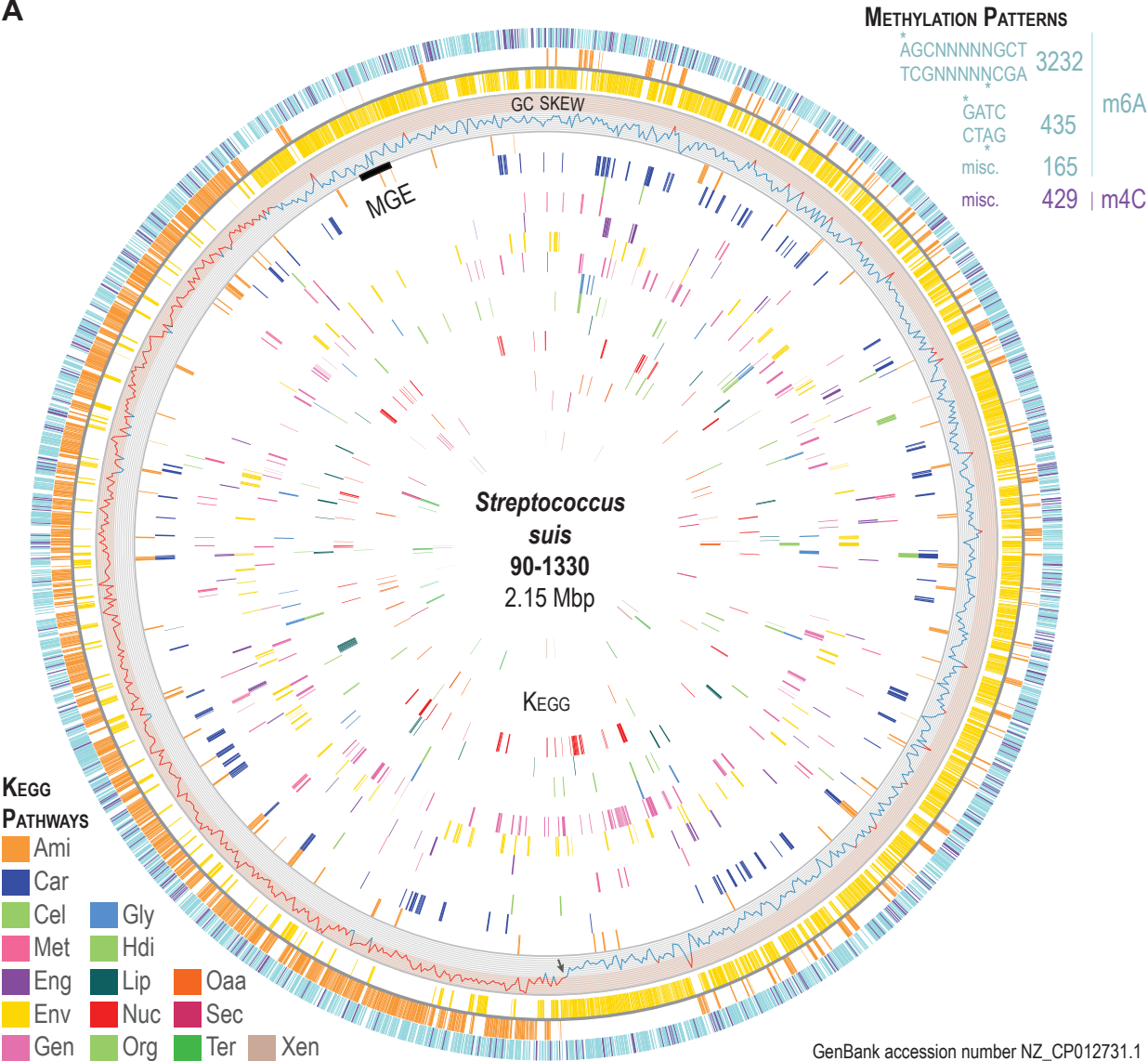

B

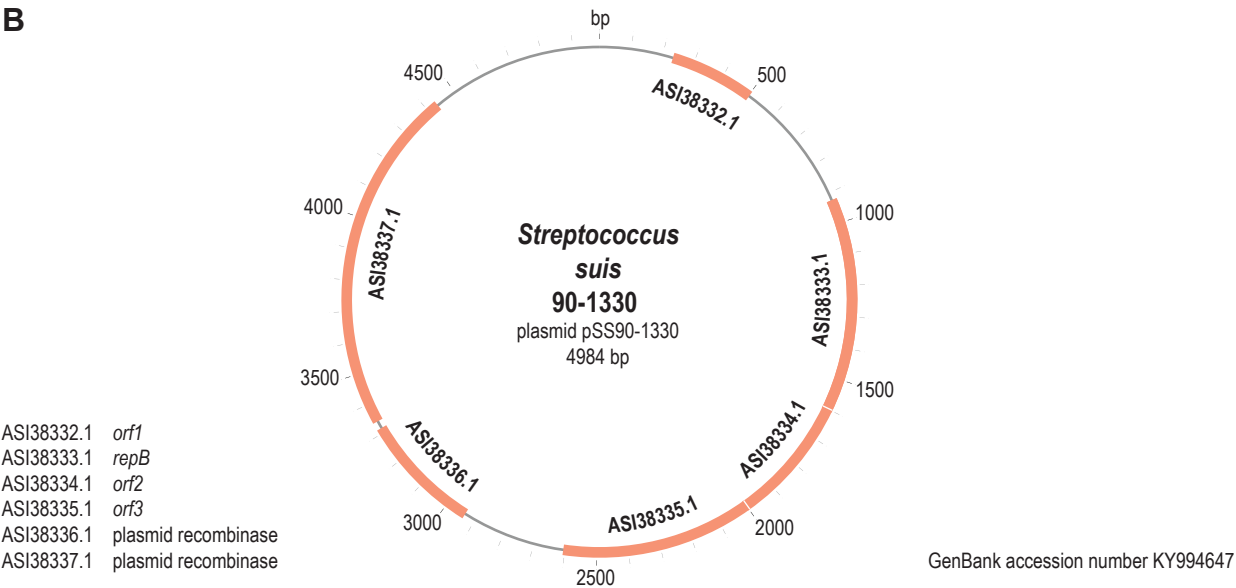

Supplement: S1 Fig — (A) S. suis 90–1330 chromosome. Genes located on the forward strand are indicated by orange boxes whereas those located on the minus strand are highlighted in yellow. Methylation patterns and GC-skew distribution are indicated in the outer and inner rings, respectively. GC-skew patterns (positive and negative values are shown as red and blue, respectively). The putative origin of replication inferred by the GC-skew analyses and the location of dnaA is indicated by an arrow. MGE; bacteriocin-containing mobile genetic element. The sixteen inner rings surrounded by the GC skew plot highlight genes of known function color-coded per KEGG pathways. From outside to inside: amino acid metabolism (orange; 126 genes), carbohydrate metabolism (blue; 246 genes), cellular processes (light green; 14 genes), metabolism of cofactors and vitamins (pink; 57 genes), energy metabolism (violet; 68 genes), environmental information processing (yellow; 151 genes), genetic information processing (pale red; 181 genes), glycan biosynthesis and metabolism (blue; 24 genes), human diseases (green; 50 genes), lipid metabolism (dark cyan, 42 genes), nucleotide metabolism (red; 99 genes), organismal systems (green; 15 genes), metabolism of other amino acids (orange; 26 genes), biosynthesis of other secondary metabolites (pink; 23 genes), metabolism of terpenoids and polyketides (lime green; 24 genes) and xenobiotics biodegradation and metabolism (brown; 22 genes). (B) S. suis 90–1330 plasmid. (PDF) [file pone.0216002.s001.pdf]

**A**

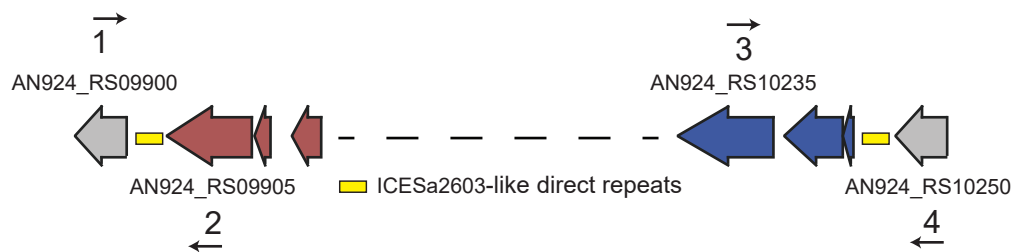

**B**

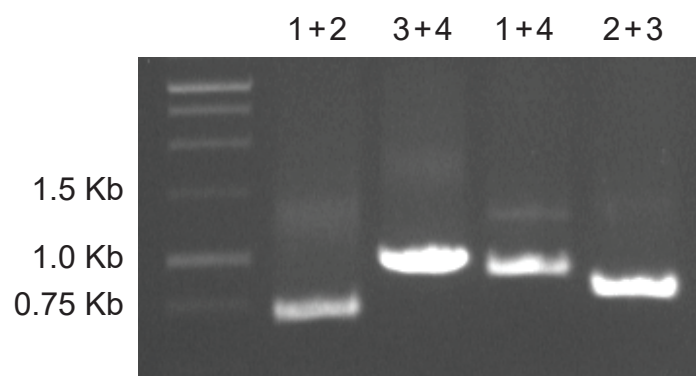

Supplement: S2 Fig — (A) Diagram of the chromosomic region contiguous to the ICE integration site. The ICESa2603-like direct repeats are represented by yellow boxes. The orientation of the primers used to detect the integrated and circular forms are indicated by thin arrows (see Material and Methods for the primer sequences): 1) AN924_RS09900 –hypothetical protein; 2) AN924_RS09905 –integrase; 3) AN924_RS10235 –replication initiator protein; 4) AN924_RS10235 –ribosomal protein L7/L12. (B) Electrophoretic analysis of the PCR products (0.8% agarose gel); primer pairs are shown above the lanes. (PDF) [file pone.0216002.s002.pdf]

**S. suis 90-1330 putative ICE**

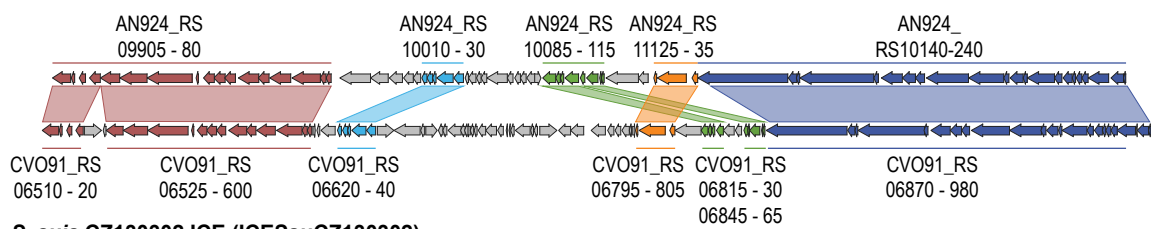

**S. suis CZ130302 ICE (ICESuCZ130302)**

Supplement: S3 Fig — Conserved blocks of genes arrayed in the same order between the two loci are highlighted by alternating colors. Locus tags are derived from accession numbers NZ_CP012731.1 (S. suis 90–1330) and NZ_CP024974.1 (S. suis CZ130302). (PDF) [file pone.0216002.s003.pdf]
